# Frequency Domain Analysis of Sensor Data for Event Classification in Real-Time Robot Assisted Deburring

**DOI:** 10.3390/s17061247

**Published:** 2017-05-30

**Authors:** Bobby K Pappachan, Wahyu Caesarendra, Tegoeh Tjahjowidodo, Tomi Wijaya

**Affiliations:** 1 Rolls-Royce @ NTU Corporate Lab, 65 Nanyang Avenue, Singapore 637460, Singapore; kbobby@ntu.edu.sg (B.K.P.); wcaesarendra@ntu.edu.sg (W.C.); twijaya@ntu.edu.sg (T.W.); 2 School of Mechanical and Aerospace Engineering, Nanyang Technological University, Singapore 639798, Singapore

**Keywords:** machining, deburring, Welch’s estimate

## Abstract

Process monitoring using indirect methods relies on the usage of sensors. Using sensors to acquire vital process related information also presents itself with the problem of big data management and analysis. Due to uncertainty in the frequency of events occurring, a higher sampling rate is often used in real-time monitoring applications to increase the chances of capturing and understanding all possible events related to the process. Advanced signal processing methods are used to further decipher meaningful information from the acquired data. In this research work, power spectrum density (PSD) of sensor data acquired at sampling rates between 40–51.2 kHz was calculated and the corelation between PSD and completed number of cycles/passes is presented. Here, the progress in number of cycles/passes is the event this research work intends to classify and the algorithm used to compute PSD is Welch’s estimate method. A comparison between Welch’s estimate method and statistical methods is also discussed. A clear co-relation was observed using Welch’s estimate to classify the number of cycles/passes. The paper also succeeds in classifying vibration signal generated by the spindle from the vibration signal acquired during finishing process.

## 1. Introduction

In machining processes, ensuring the quality of a finished product is crucial and with advances in manufacturing technology, a need exists to integrate in-process monitoring technology into the production environment, so as to avoid manufacturing induced anomalies [1,2,3]. Advanced process monitoring technology coupled with an intelligent decision making support system can reduce the time taken to otherwise perform rework on finished components with defects. This will save costs and also reduce the dependency on skilled operators. A report released by the Federal Aviation Authority (FAA) in partnership with the Aerospace Industries Association (AIA) Rotor Manufacturing (RoMan) project team in the year 2006, stresses the importance of incorporating advance process monitoring and control technology in manufacturing processes especially for critical aerospace components [4]. Process monitoring is generally classified into direct and indirect methods. In direct method, the quantity of the output variable is measured or monitored directly whereas in indirect method, the output variable is deduced through monitoring the quantity of process variables such as vibration, speed [5]. While direct method is known for its accuracy, indirect method is widely accepted since they are more realistic to be implemented in an industrial environment as the cost incurred is comparatively less than direct method. Indirect process monitoring is performed by capturing these process variables with the means of sensor systems e.g., accelerometer, dynamometer, temperature sensor etc. Standard data acquisition (DAQ) systems are then used to acquire, sample and log the data. The data is further analysed to identify any significant and/or persisting trend/patterns. Subsequently, the analysed data can be used to deduce the required output variable. This analysis can also be performed real-time making indirect method more efficient than direct methods.

Indirect process monitoring requires an intelligent method to analyze big data generated by the sensors. An important step involved in intelligent data analysis is to identify the signal signature and used the signatures in the learning method. The signatures of the raw vibration signal can be obtained by the feature extraction methods in either time-domain, frequency-domain or time-frequency domain [6]. A number of literatures has presented the learning methods for mechanical sensor data analysis especially for vibration signals [7,8,9]. For example, Lei et al. [7] present an analysis of mechanical big data using unsupervised feature learning method. The method consist of two learning stages i.e., sparse filtering with two-layer neural network and softmax regression. Recently, the concept of intelligent method has also applied in manufacturing process for indirect surface quality monitoring [10,11,12].

This paper focuses on identifying a relevant signature in frequency domain that gives direct information of the progress of machining process. In the following sections, an overview of sensor-based monitoring and signal processing methods in machining applications is presented. Section 3 and Section 4 cover the experiment setup under which this research work was performed, the results obtained and inferences deduced from the results.

## 2. Process Monitoring in Machining

**Figure 1:**
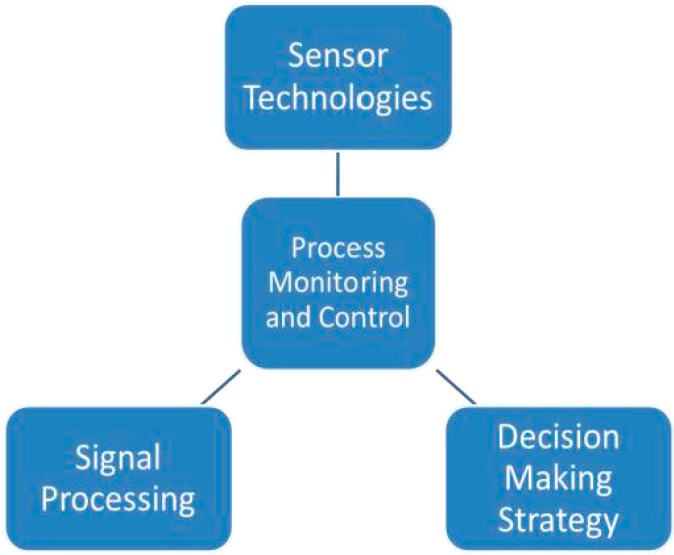
Building blocks of machining process monitoring (indirect).

Machining is the term used for manufacturing processes that involves varying ranges of material removal rate (MRR). Machining performed with hard tools has higher material removal rates compared to finishing processes, wherein compliant abrasive tools like brushes/belts are used. In a machining process monitoring system as shown in Figure 1 the cutting region involves several process variables, such as vibrations, cutting forces, acoustic emission, temperature and surface finish. The various factors that influence these process variables include the state of the cutter/tool, coolant flow, chip packing and other material removal process conditions.

By using appropriate physical sensors, the variable that needs to be measured can be continually monitored and variations can be logged [13]. The data acquired is processed with the aim to identify patterns, trends or abnormal process conditions. Further analysis is performed on the acquired data with the help of machine learning algorithms such as neural networks and fuzzy logic [14]. Upon detection of any process related information or process faults, the information is communicated either to the operator or fed directly to robot controller to take relevant corrective/adaptive actions.

A majority of past research works on process monitoring was performed on processes involving hard tool, e.g., milling, turning. In most cases the focus of the work is inclined towards tool condition monitoring [15]. The focus of this research work is to identify information that has some co-relation with the completed number of passes in a robot assisted finishing process. This was achieved by analyzing the corresponding magnitude levels of signal in frequency domain from different passes and belonging to a fixed frequency band. Further details on the experiment are mentioned in Section 3.

### 2.1. Sensors and Signal Processing

**Figure 2:**
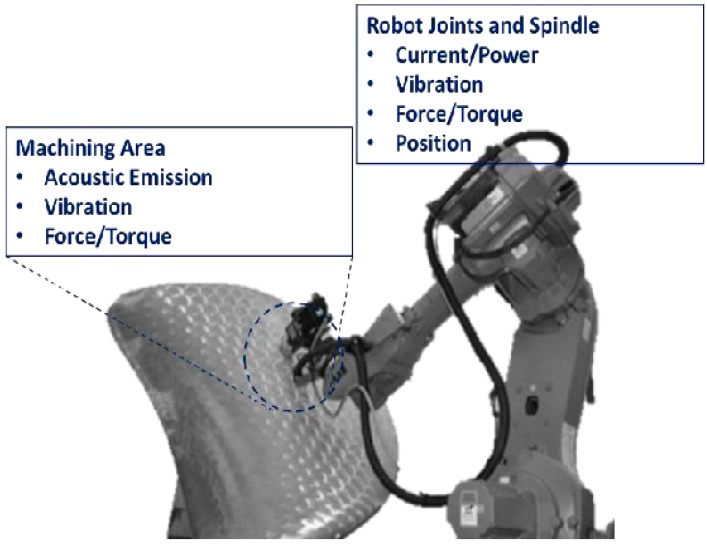
Measurable phenomena in machining environment.

Some potential measurable process phenomena in a robotic machining environment are shown in Figure 2. Power and current flow of the spindle delivers the required cutting force. Hence monitoring the power intake and current flow in motors that drive the spindle can be used to understand the MRR [16,17]. However, in robot assisted finishing processes, monitoring and implementing spindle drive control is impractical due to complex architecture compared with traditional milling or turning machines or numerical control (NC) machines. As shown in Figure 2 monitoring the measurable phenomena which are closer to the machining area is a better alternative to understand and analyze the nature of the process. This include acoustic emission (AE), force/torque exerted by the tool on the workpiece, vibration and spindle motion displacement. 

Signal signatures often consist of embedded information which can be co-related to a process variable itself. Signal processing plays a pivotal role in performing this task by extracting the relevant signatures and also for identifying trends/patterns. A wide range of signal processing techniques exist and choosing an ideal technique relies heavily on the type of application.

The acoustic emission (AE) sensor is known for its susceptibility towards high frequency signals (above 20 kHz) and clearly seems to be a favorite choice in most machining process monitoring applications. Using an AE sensor also reduces the requirement to perform further signature extraction as the AE signal has a relatively higher signal to noise (S/N) ratio and improved frequency response for high frequencies [18]. However, in applications that involve a low material removal rate (MRR), the magnitude of elastic waves produced by tool-workpiece interaction is much lower compared to processes like turning, milling, drilling, etc. In such cases, other sensing systems must be relied upon to give meaningful information regarding the process (e.g., vibration sensor). Table 1 gives a summary of past literature on types of sensing systems and signal processing used, classified based on monitoring aspect. An exhaustive review on process monitoring including the types of sensors used and signal processing methods is given in [6]. In our experiments we have employed a tri-axial accelerometer to capture vibration data.

### 2.2. Frequency Domain Analysis

Signal processing techniques can be broadly classified as time domain and frequency domain. Several studies have used both techniques in applications involving tool wear/breakage detection and indirect surface integrity detection. For instance, in [19], kurtosis (time domain) and frequency domain analysis is used successfully to understand tool properties in a drilling process. In time domain analysis, statistical methods are used to distinguish persisting patterns/trends. This includes skewness, kurtosis, co-relation coefficient, etc. In frequency domain analysis, a captured signal is analyzed in the frequency domain and changes to individual frequency components are often indicative of the changes in process variables. 

**Figure 3:**
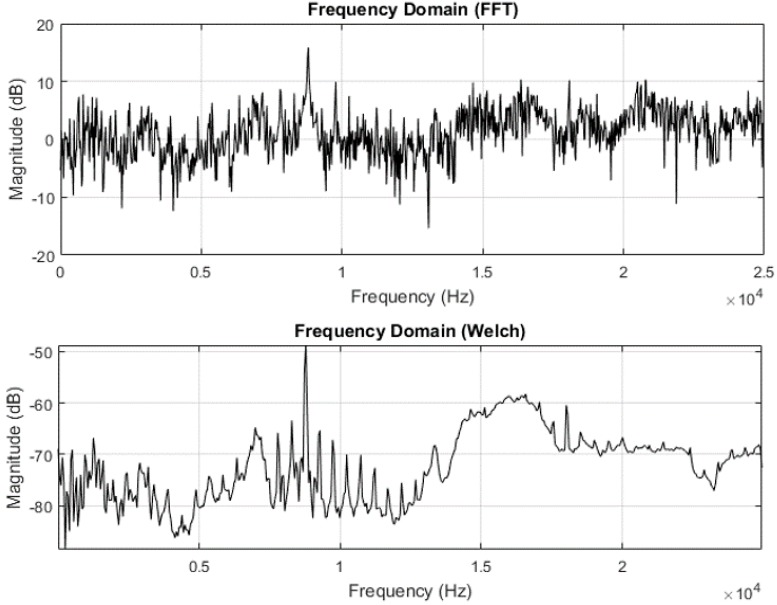
Frequency domain analysis using FFT and Welch estimate.

Frequency domain analysis also helps to visualize the effect of noise filtering and various other windowing and filtering techniques. As the sampling frequencies in this experiment fall in the range of 40–51.2 kHz, performing time domain analysis has proved to be challenging due to the size of the data captured and hence frequency domain analysis is effective to understand process characteristics. Signal power also contains pertinent information regarding the source of signal generation. Conventionally, fast Fourier transform (FFT) has been in use to determine power spectra. In stochastic processes, performing FFT will not be useful to segregate the noise embedded in the signal, as the frequency of interest might be eclipsed by the sidelobes created by higher frequency content. Hence some averaging needs to be performed to increase the S/N ratio and to also make all embedded frequencies visible. Welch’s power spectrum estimate essentially does this by calculating the power spectrum using FFT, coupled with averaging. This helps to minimize the signal power caused by random variations. FFT do not account in for discontinuities between successive periods as the data captured is assumed to be of a single period of a periodically repeating waveform and this phenomenon is referred to as spectral leakage. Figure 3 below shows this effect demonstrated on a set of data recorded from an accelerometer during a finishing process. As shown in Figure 3, the power spectrum calculated using Welch’s estimate contains comparatively lesser clustered information than with the FFT method. Applying Welch’s estimate method thus also helps to reduce spectral leakage and reduces the effect caused by undesired frequencies.

Welch’s method to compute PSD is performed by dividing time series data into segments that are successive and averaging the periodograms of each segments or frames. Consider $x_{m}(n)$ to be the input signal where m=0, 1, …,K, K=total number of frames and n=0, 1, …, M−1, periodogram of $m^{th}$ frame is given by:

(1) $$Px_{m},\;M(k)=\frac{1}{M}{\left|\sum_{n=0}^{N-1}x_{m}(n)\;e^{-\frac{2\pi nk}{N}}\right|}^{2}$$

then Welch’s power spectrum estimate is computed as:

(2) $$S_{x}(w_{k})=\frac{1}{K}\sum_{m=0}^{K-1}P\;x_{m},\;M(k)$$

Upon analyzing the power readings of certain frequency components, it was noted that the changes observed corresponded to the completed number of passes. Analyzing the frequency components of the vibration signal is imperative to finishing processes as the fundamental frequency and its harmonics contain coherent information which can be attributed to spindle behavior and also the finishing of the component. Shop floor operators require systems that are less sophisticated and adopting a frequency domain analysis method gives that flexibility as opposed to other machine learning algorithms or statistical methods. Welch’s estimate method is preferred as an easier method to implement in such cases as it gives a more visual means of interpretation.

### 2.3. Event Classification

**Figure 4:**
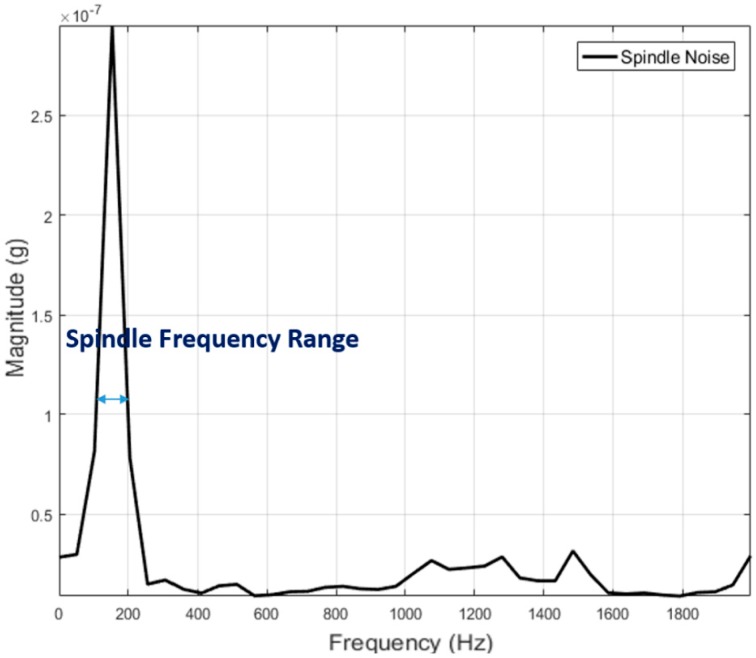
Vibration signal from spindle.

Extracting coherent information from frequency domain signal requires proper understanding of the process. The frequency content shown in the spectrum often has direct connection to process associated events. It is also crucial to separate low frequency noise created by voltage fluctuations. To address this, a band pass filter with low and upper cut-off frequency of 100 Hz and 20,000 Hz respectively was employed during data acquisition. In finishing processes, another major source of vibration signal is the finishing tool. The drive system for this tool acts as source for different frequency content. Hence identifying as much as background information regarding the process and equipment involved, is vital for interpreting frequency domain data. The spindle mounted on to robot end-effector is controlled by a variable frequency drive (VFD) capable of delivering an output frequency range up to 400 Hz or 24,000 RPM. This VFD can be programmed to operate at required RPM and in our experiment, the frequency was set at between 150–170 Hz to drive the tool at an RPM of 10,000. If information pertaining to finishing alone has to be derived, all other frequencies associated to process under consideration must be identified. To accomplish this, vibration signal generated by the spindle when there is no finishing is under progress was acquired. Figure 4 shows this signal as acquired from the accelerometer. The Figure 4 shows a dominant peak at 155 Hz and is interpreted as the frequency generated by the rotating tool.

This interpretation helps to identify frequencies corresponding to finishing process and allows one to co-relate effectively with the event needed to be classified. Latching a co-relation algorithm on spindle frequency is unreliable as the spindle performance may vary non-linearly or remain the same throughout. This uncertainty left unaccounted for will cause the classification algorithm to give an erroneous output.

**Figure 5:**
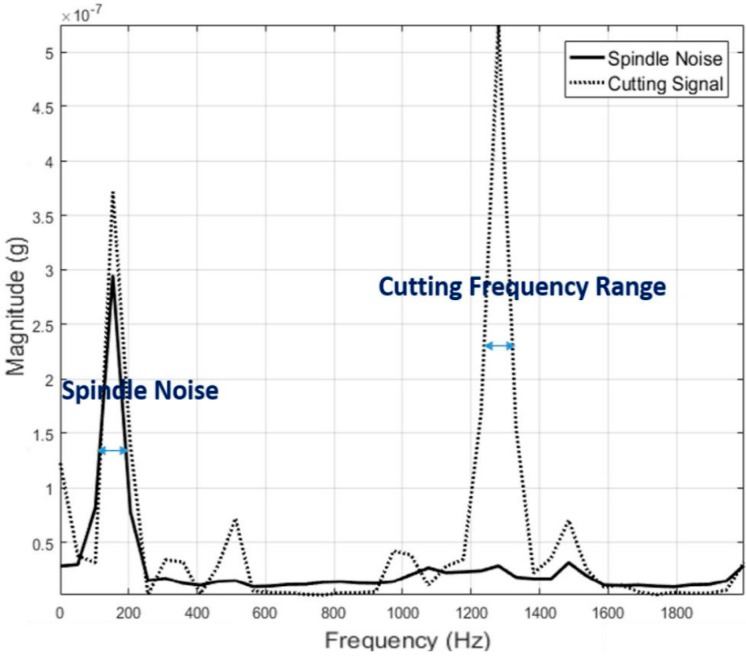
Vibration signal from finishing process overlapped on spindle noise.

After identifying known frequency ranges, co-relation can be performed with remaining dominant frequencies. In Figure 5, a vibration signal acquired from a spindle is shown overlapped on the signal acquired when finishing is under progress. 

**Figure 6:**
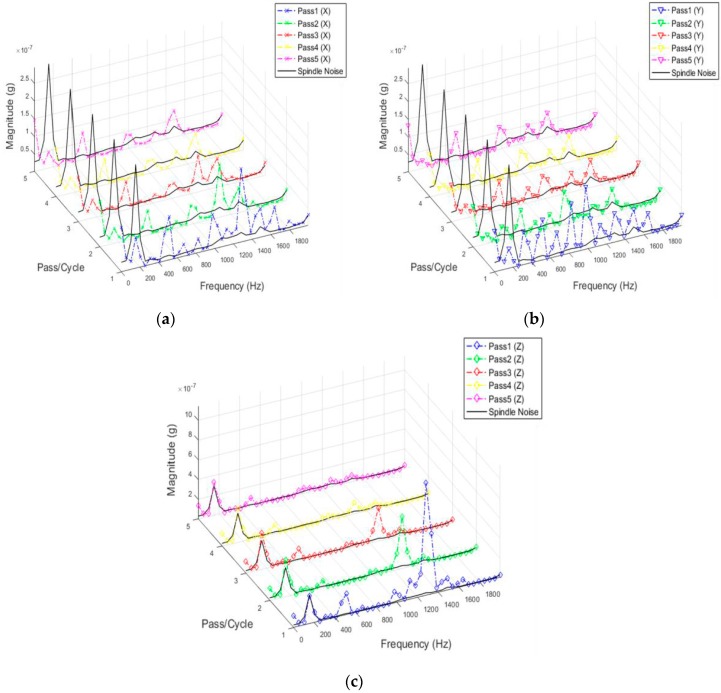
(**a**) Vibration signal (*X*) from each pass/cycle overlapped on spindle noise; (**b**) Vibration signal (*Y*) from each pass/cycle overlapped on spindle noise; (**c**) Vibration signal (*Z*) from each pass/cycle overlapped on spindle noise.

In the vibration signatures acquired during finishing process, the frequency ranges between 1200 and 1400 Hz showed considerable activity when compared to vibration signal from the spindle. This observation was validated by plotting the vibration signals from every other pass and the results are shown in Figure 6. The identified frequency range is extracted afterwards to search for peculiar behaviour with completed number of passes/cycles. Specifically, the vibration signature at 1.297 kHz showed a good correlation with the completed number of passes/cycle and this feature is used for co-relation and event classification in this research work. In any sensor-based process monitoring system, which needs to use high sampling rates, performing spectral analysis on acquired signals has thus proven beneficial to classify events occurring during the process and furthermore, it can be used as an input for machine learning purposes.

## 3. Experiment Setup

**Figure 7:**
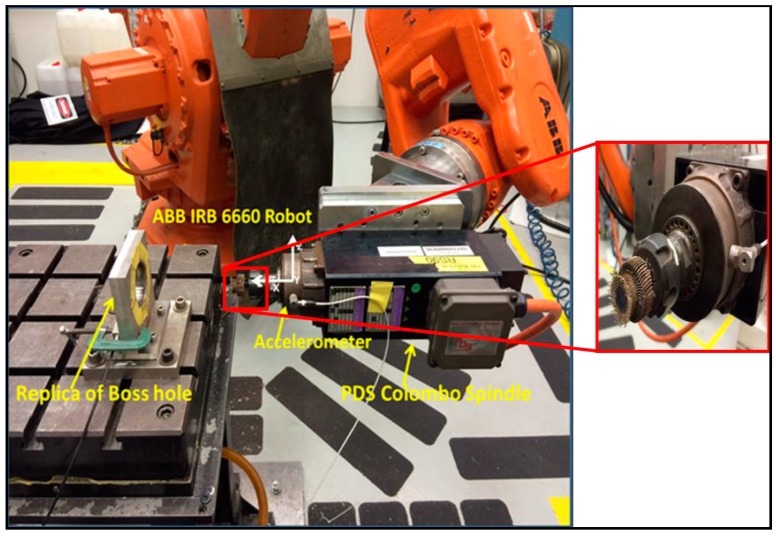
Trial and experimental setup.

The experiment setup (Figure 7) comprises of an ABB IRB 6660 machining robot and PDS colombo spindle. The representative work coupon used for machining is a boss hole of a combustor casing and the objective is to remove the burrs until a chamfer is developed. As mentioned in Section 2, vibration signatures were measured using a Kistler 8763B (IEPE) tri-axial accelerometer. The RPM of the spindle was kept constant at 10,000 RPM and feed rate at 30 mm/s. The data acquisition devices used were NI cDAQ-9184 and NI 9234 IEPE.

### 3.1. Data Analysis and Results 

**Figure 8:**
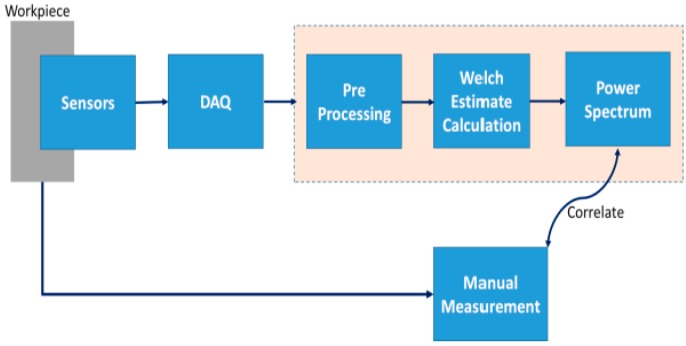
Process flow diagram.

Data was captured at a sampling rate of 40 kHz and for computational ease, pre-processed to 1000 samples per each iteration of Welch’s estimate calculation. A total of 12 experiment sets was collected with eight being used for offline analysis and co-relation and another four for validation. After each cycle of machining, the chamfer length was manually measured using laser measuring device. In offline data analysis, co-relation between the measured values and variations in estimated power spectrum is analyzed. The co-relation between the power spectrum and number of cycles was subsequently validated in real-time. Figure 8 shows the process flow of how experiments were conducted. 

**Figure 9:**
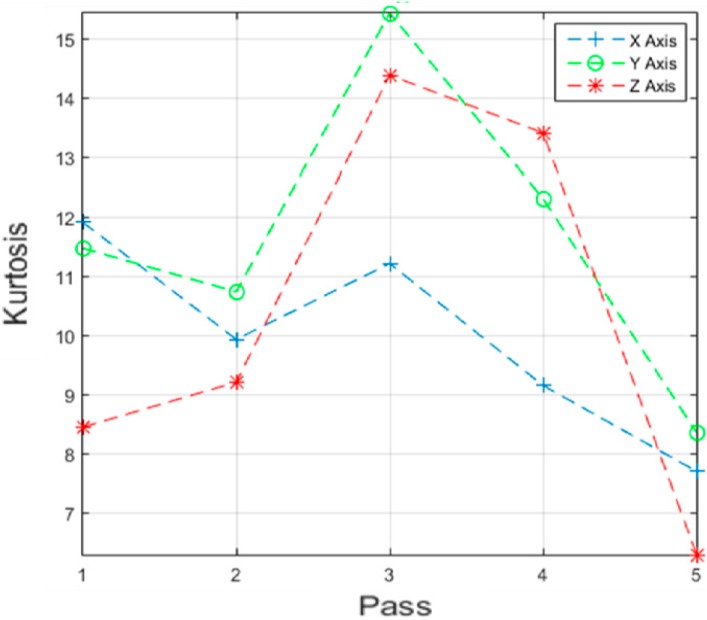
Kurtosis vs. number of passes.

**Figure 10:**
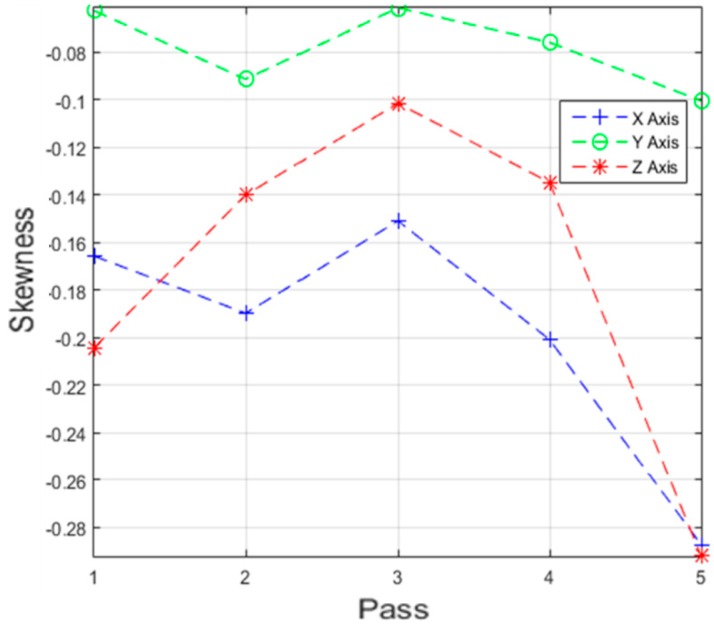
Skewness vs. number of passes.

**Figure 11:**
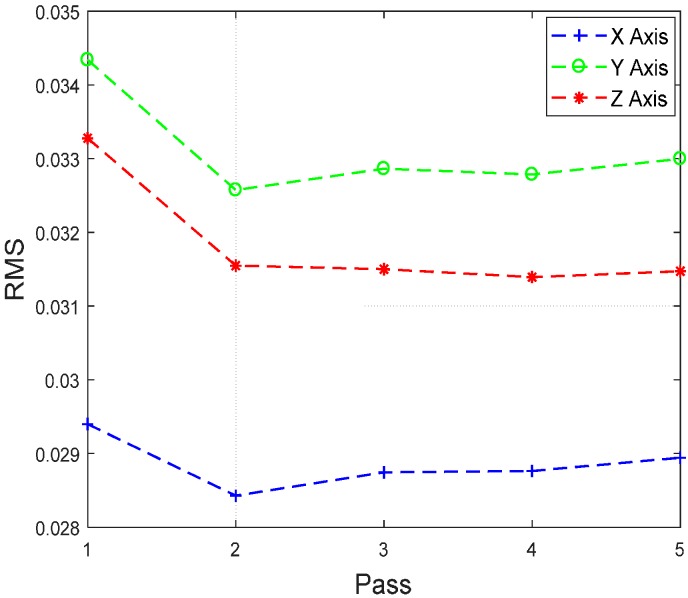
RMS vs. number of passes.

**Figure 12:**
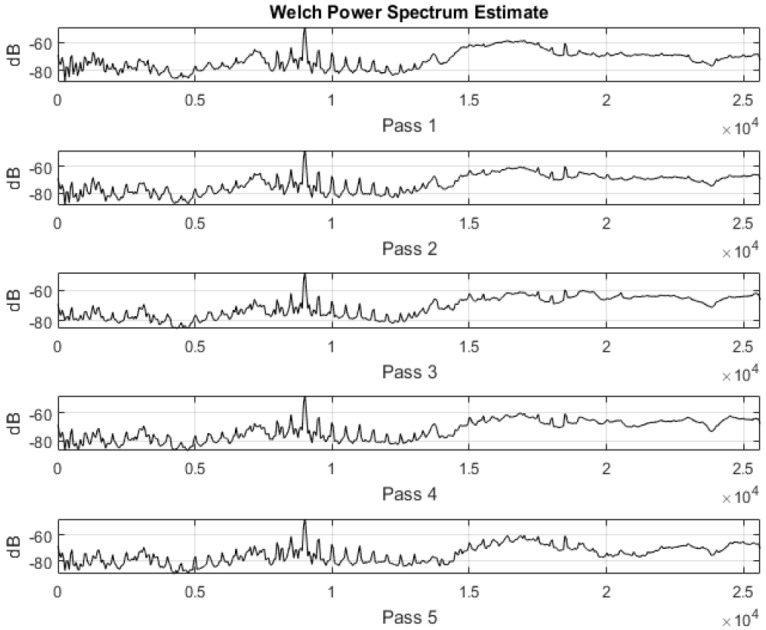
Welch’s power spectrum estimate for each pass/cycle.

**Figure 13:**
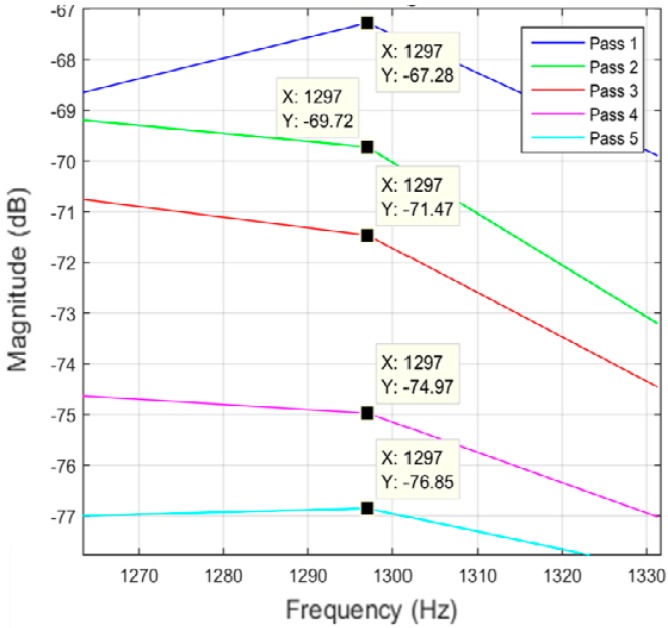
Vibration signal in *X* direction.

**Figure 14:**
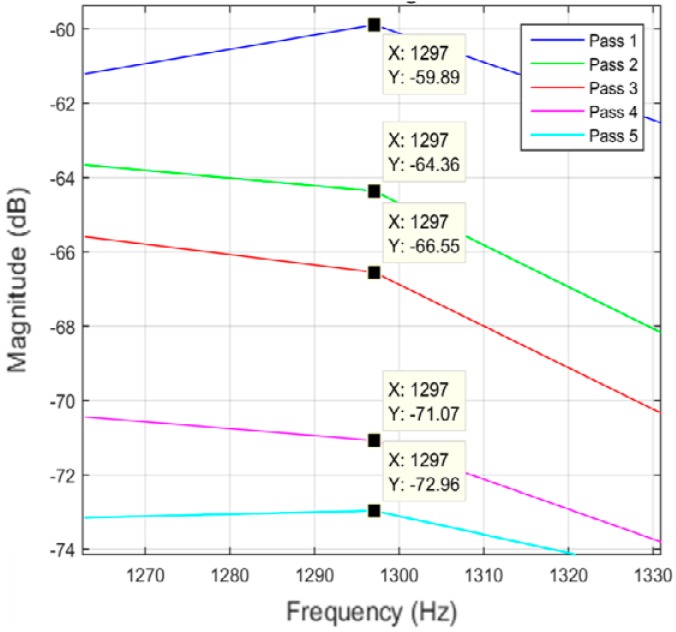
Vibration signal in *Z* direction.

**Figure 15:**
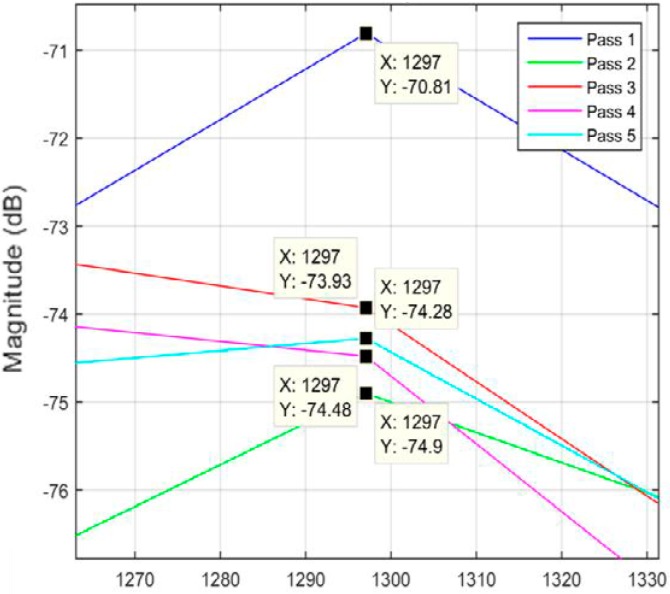
Vibration signal in *Y* direction.

Different data analysis techniques were used on acquired data sets. For instance, kurtosis, skewness and root mean square (RMS) values for respective passes were calculated but it failed to show any consistent trend or pattern with increasing number of passes. The skewness, kurtosis and RMS calculation was performed on the entire time-series data of each pass and the results obtained are shown in Table 2. It can be noted from the numbers that a pattern or trend is not obvious. This can also be also understood from Figure 9, Figure 10 and Figure 11. The result obtained after performing a Welch’s power spectrum estimate is shown in Figure 12. Figure 13, Figure 14 and Figure 15 shows the Welch’s power spectrum estimate at 1.297 kHz. As shown in Figure 13, Figure 14 and Figure 15, the power values of vibration signal decreases with respect to the increasing number of passes. The trend here when compared with kurtosis and skewness values is more obvious to the naked eye. The decrease in signal magnitude is indicative of the strength of signal. It is also understood that the decrease in strength of signal is caused due to the smoothening of edges of boss hole with increasing number of passes (Table 3 shows the increase in chamfer radius with each different pass).

**Figure 16:**
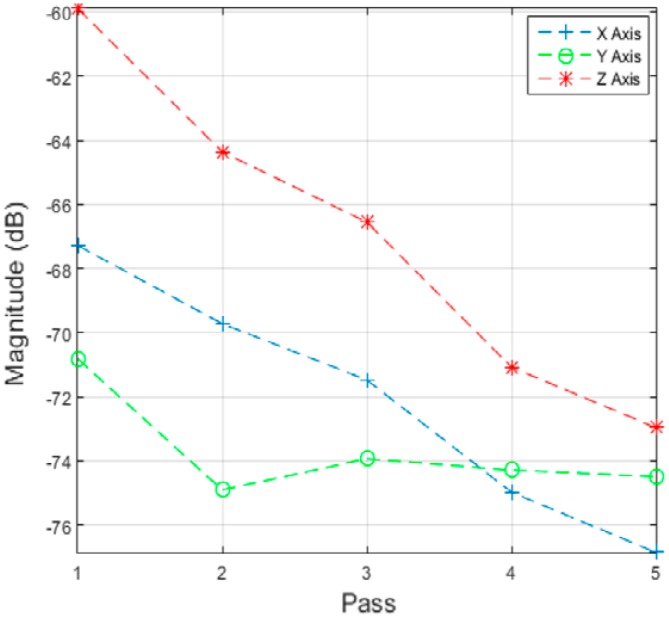
Signal magnitude vs. number of passes/cycle.

From the vibration signatures, it can be concluded that a co-relation exists with the different number of passes and the PSD at 1.297 kHz. This signal as explained in Section 2.3 is associated with the finishing process itself. The spindle RPM is controlled by a variable frequency drive (VFD) controller and the frequency is fixed at 165 Hz. This is however with the exception of *Y* axis measurement and is caused due to the orientation and placement of the sensor. Figure 16 shows the correlation between magnitudes of the captured signal with increasing number of passes. From the Figure 16 it can be seen that magnitude of vibration signal in *Y* axis is relatively low as compared to *X* and *Z* axis measurement which further confirms the assumption on sensor location aspect.

### 3.2. Sensor Placement Effect on Vibration Signal

**Figure 17:**
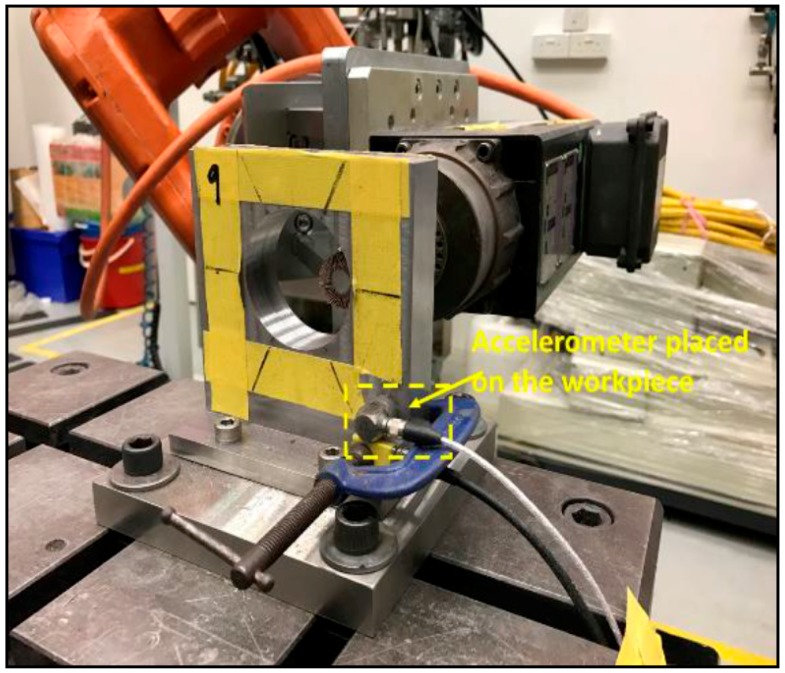
Accelerometer attached to workpiece.

The results obtained and described in previous sections is characteristic to the sensor location when it is placed on the tool (see Figure 17). To understand the effect of spindle noise on acquired signal, further data analysis was performed with sensor placed at alternate locations. Another reason to consider alternate sensor placement location is also to account for minimized distractions during actual production scenario. With sensor placed on the tool, there is a risk of wire entanglement as well as constraints with robot movement. 

**Figure 18:**
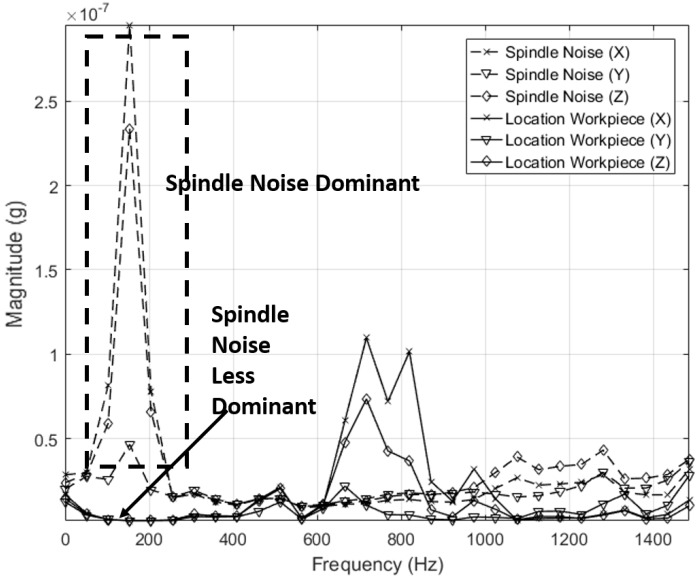
Vibration signal when sensor is placed on (1) Tool and (--) (2) Workpiece (-).

As the intention of the research undertaken is eventual deployment in production, sensor placement issues must also be addressed. As such, the vibration signal was acquired with a sensor placed on the workpiece (Figure 17). The vibration signal from the accelerometer was acquired at a sampling rate 40 kHz. Similar signal pre-processing methods were used with a previous experiment set up where the sensor is placed on the tool. After acquiring the vibration signal, Welch’s estimate method was used to visualize the signal in the frequency domain. The Welch’s estimate of the signal is shown in Figure 18. 

Figure 18 also shows the vibration signature obtained when the sensor was placed on the tool so that the effect of sensor placement can be studied. From Figure 18 it can be understood that spindle frequency content is less dominant in the vibration signal acquired when the sensor is placed on the workpiece. In the signal acquired when the sensor is placed on the tool, the spindle frequency content is dominant with the frequency previously established as associated to the spindle speed being the highest. This further validates the finding that frequency generated by the spindle is within the range of 150–200 Hz and hence cannot be relied upon for co-relation. 

**Figure 19:**
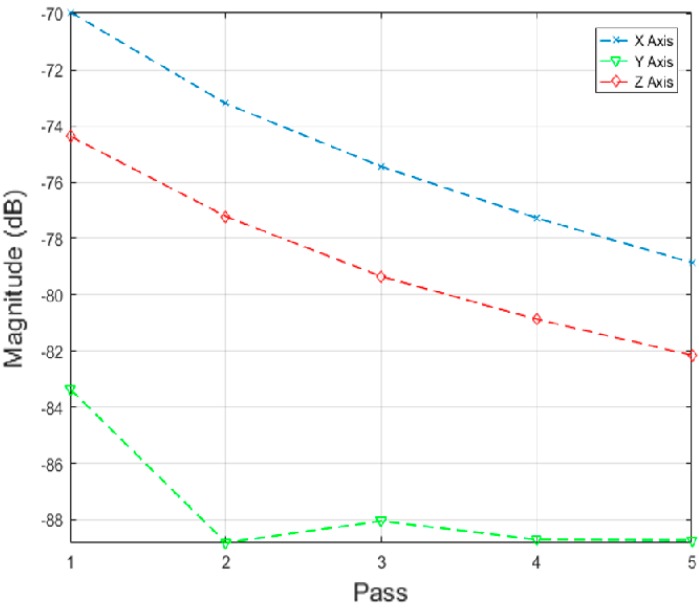
Signal magnitude vs. number of passes/cycle.

The vibration signal when finishing is in progress was also acquired with the sensor located on the workpiece and a similar co-relation as that shown in Figure 16 was also obtained. This result is shown in Figure 19. It is worth noting that a similar pattern was found with the vibration signatures of different passes, irrespective of the sensor location. With the exemption of the *y* axis signature, *x* and *z* axis signature showed linear co-relations with the number of completed passes. This is good in terms of co-relation perspective, as it further establishes the methodology used here for event classification.

### 3.3. Fuzzy Inference System (FIS)-Based Event Classification

Real time monitoring of surface finish quality in the aerospace manufacturing industry requires uncomplicated yet reliable feature extraction methods to attain an accurate prediction. This study focused on a feature extraction and classification method that is applicable to the real time monitoring based on the LabVIEW and NI platform. Features in the time-domain analysis such as skewness, kurtosis and RMS are analysed and compared to the Welch’s power spectrum estimate. The features were extracted from twelve vibration datasets that are acquired during lab experiments (a deburring process). The deburring processes were conducted on each work coupon in five sequential stages, namely pass 1 to pass 5. Each pass contains one vibration dataset that is acquired during one rotation (360°) of a circular abrasive tool on the edge of a boss hole replicated from a jet engine combustor casing. It is presumed that the vibration signal of pass 1 to pass 5 has event characteristic because it is understood that the different in signal characteristics are due to the smoothening of the edges of the boss hole with increasing number of passes. Therefore the features extracted from each dataset should also correspond to the number of passes. The extracted skewness, kurtosis and RMS from different passes has been presented in Figure 9, Figure 10 and Figure 11. The trends of skewness, kurtosis and RMS feature were not obvious compared to the Welch’s feature which is shown in Figure 13, Figure 14 and Figure 15 for vibration signals on three different axis. The Welch’s feature were then employed in the event classification of the deburring process.

Due to the highly stochastic behavior of vibration data that are acquired from deburring processes, the trends of the Welch’s feature were obvious for seven out of the twelve datasets. Among the seven datasets, six datasets were used for training and one dataset for testing. The training datasets are presented in Table 4. The maximum, median and minimum magnitude value of each pass are calculated and used to build the triangle membership function of FIS Sugeno type. The overall maximum and minimum value for all datasets is −84.7 dB and 70.25 dB. These values are used as the range to build a triangle membership function of FIS Sugeno type.

**Figure 20:**
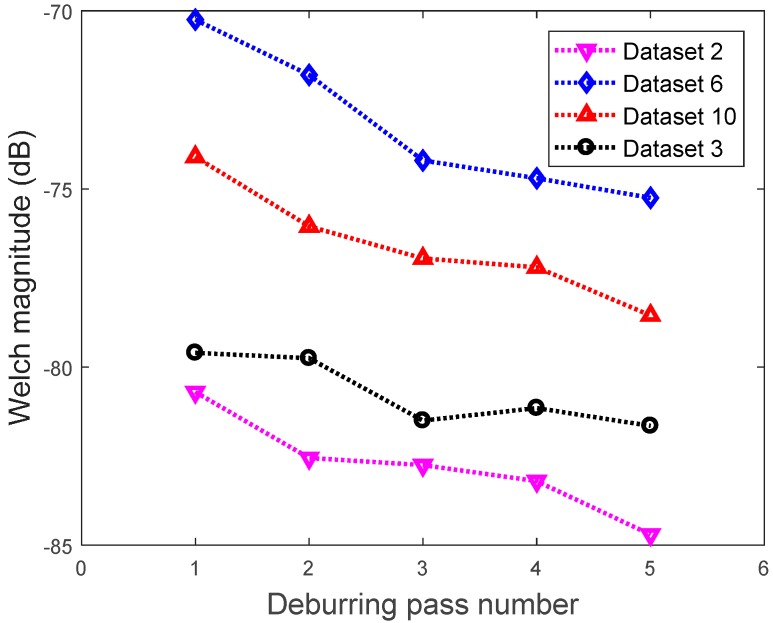
The representative plot of four datasets.

The representative trends of prominent and inconspicuous Welch’s features are presented in Figure 20. For dataset #2, #6 and #10, the magnitude of the Welch estimate at a frequency of 1.297 kHz are decreasing gradually, which corresponds to the number of passes. However, the dataset #3 did not decrease gradually as shown in the black dotted line. An anomaly occurred on pass #4 where the magnitude of 1.297 kHz was higher than the magnitude on pass #3. This was considered as an inconspicuous trend of the Welch’s features. Further study will be conducted on this phenomenon, however, the authors assumed that in the present study this is due to some stochastic behavior of the accelerometer data.

**Figure 21:**
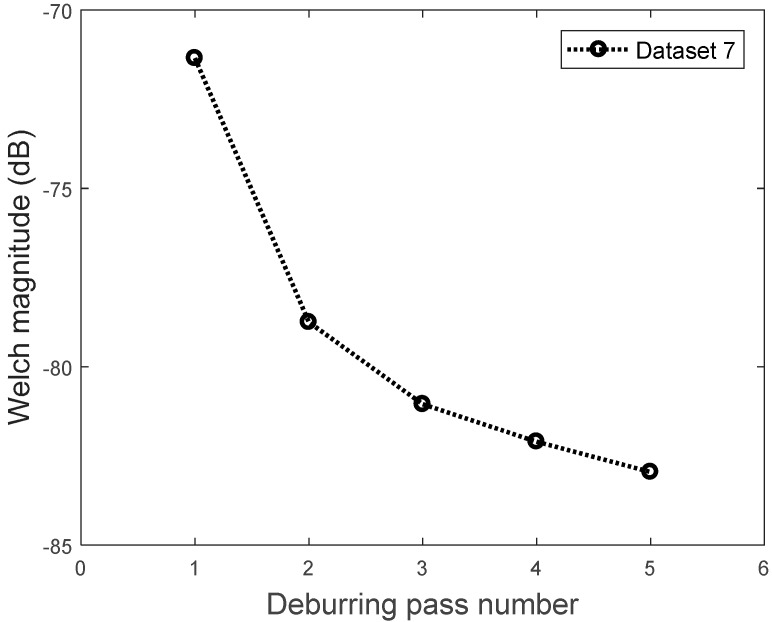
The test dataset.

For simplicity for LabVIEW real time monitoring, Fuzzy Inference System (FIS) designer apps from MATLAB is used in the present study for classification. The test set used was the dataset #3 as shown in Figure 21. The magnitude changes in dataset #3 was the ideal result for deburring stages where the material removal of the first deburring step is much higher than the next stages. This is shown in Figure 21 where the amplitude difference between pass #1 to pass #2 was higher than the next stages. As the deburring pass number increases, the material removal rate was also getting smaller. This is indicated in the last two passes in Figure 21.

**Figure 22:**
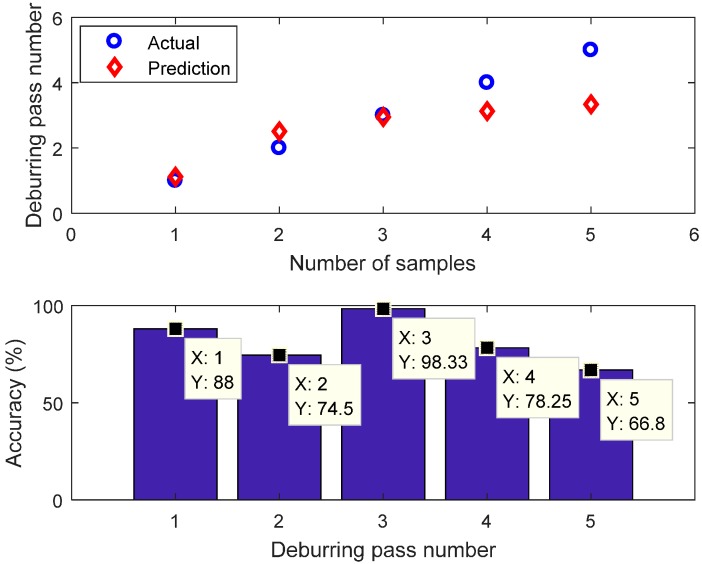
FIS Mamdani prediction.

**Figure 23:**
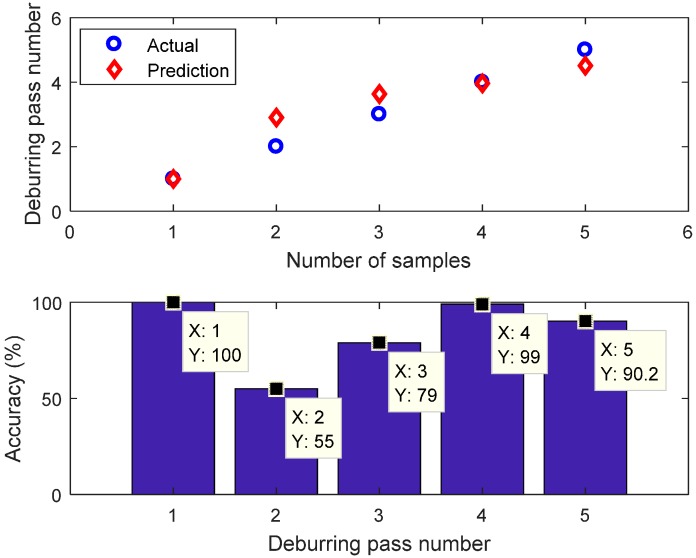
FIS Sugeno prediction.

Two types of FIS, that is Mamdani and Sugeno, were used and compared. This paper used number of passes as the classification label. The test dataset was fed to the Mamdani and Sugeno FIS model. The FIS input was the magnitude of the component at 1297 Hz of the Welch’s power spectrum estimate (in dB) and the output of classification is the label (number of passes). The prediction results of FIS Mamdani and Sugeno are presented in Figure 22 and Figure 23, respectively. The local accuracy of FIS Mamdani prediction is 88%, 74.5%, 98.33%, 78.25%, and 66.8% for pass #1, #2, #3, #4, and #5, respectively. The overall accuracy for FIS Mamdani prediction is 81.18%. In addition, the local accuracy of FIS Sugeno is 100%, 55%, 79%, 99%, and 90.2% for pass #1, #2, #3, #4, and #5, respectively. The overall accuracy for FIS Sugeno prediction is 84.64%.

## 4. Conclusions

The focus of this research work was to explore the relationship between captured vibration signals and the progress of an actual finishing process. The experimental results establish a linear co-relation between vibration signals with completed number of passes. The effect of sensor placement upon the vibration signals was also understood and the frequency range associated with spindle activity was identified. One major limitation which the authors noted during this research work is that the results obtained were dependent on the training experiments and hence applying the classification technique to other finishing processes may not yield the same expected outcome. This will be the focus of future research directions; to validate and set up a similar classification technique across other finishing processes like polishing. 

Spectral analysis proved to be a viable solution for performing this task and is seen as a promising technique to be implemented in real-time applications involving high sampling frequencies. The advantage seen here is that analyzing a particular frequency component relieves the need of bulk data processing as opposed to statistical methods wherein packets of data needs to be computed to understand the co-relation between different statistical attributes with the number of passes completed. Besides, the Welch spectrum estimate showed a significant co-relation with the completed number of passes as opposed to time-domain features like kurtosis and skewness. 

This research work was conducted primarily to understand the possible co-relation between sensor signal features and the progress of the finishing process. The co-relation observed will be integrated to the robotic finishing software environment used for tool path programming and will serve as a visual aid to shop floor operators enabling them to monitor the progress of the finishing process. In the next phase of the project, the signature identified as a classifier will subsequently be used as an input parameter for machine learning algorithms. Additionally, a control strategy could be deployed with a feedback loop in the robot control system to dynamically adjust the process variables to compensate for any unexpected behavior.

## Figures and Tables

**Table 1:** Process monitoring using sensors classified based on the monitored variable.

Monitoring Aspect	Sensor System Used	Signal Processing	Process
**Process**: Faults and Conditions, Chatter	AE, Vibrations, Cutting Force	Frequency domain analysis, Wavelet transform, Statistical	Milling, Turning, Other
**Tool:** Wear, Breakage	AE, Vibrations, Cutting force, Camera	Frequency domain analysis, Wavelet transform, Image analysis	Milling, Band sawing, Broaching, Turning, Tapping
**Surface Quality:** Surface finish and roughness, Surface geometry	AE, Vibrations, Cutting force	Frequency domain analysis, Statistical	Milling, Turning, Hand Machining
**Other:** Spindle, Surface integrity	Vibrations	Frequency domain analysis	Turning

**Table 2:** Skewness, kurtosis and RMS of vibration signal from 3-axes accelerometer.

Pass	Skewness			Kurtosis			RMS		
	*X*	*Y*	*Z*	*X*	*Y*	*Z*	*X*	*Y*	*Z*
1	−0.16	−0.06	−0.20	11.91	11.47	8.40	0.0294	0.0343	0.0333
2	−0.18	−0.09	−0.14	9.93	10.73	9.20	0.0284	0.0326	0.0315
3	−0.15	−0.06	−0.10	11.21	15.43	14.38	0.0287	0.0329	0.0315
4	−0.20	−0.07	−0.13	9.15	12.30	13.40	0.0288	0.0328	0.0314
5	−0.28	−0.10	−0.29	7.70	8.36	6.29	0.0289	0.0330	0.0315

**Table 3:** Increase in chamfer radius.

Pass	Chamfer Size (mm)
	(Initial: 0.84 mm)
1	0.956
2	1.028
3	1.083
4	1.098
5	1.118

**Table 4:** Training data for FIS model.

Data Number	Magnitude (dB)				
	Pass 1	Pass 2	Pass 3	Pass 4	Pass 5
Dataset 2	−80.7	−82.55	−82.75	−83.2	−84.7
Dataset 3	−79.6	−79.75	−81.5	−81.15	−81.65
Dataset 4	−80.25	−80.7	−83.2	−83.85	−84.6
Dataset 6	−70.25	−71.8	−74.2	−74.7	−75.25
Dataset 8	73.3	−74.35	−76.7	−77.9	−79.1
Dataset 10	74.1	−76.05	−76.95	−77.2	−78.55
Min	−70.25	−71.8	−74.2	−74.7	−75.25
Median	−76.85	−77.9	−79.23	−79.53	−80.38
Max	−80.7	−82.55	−83.2	−83.85	−84.7
